# Distinct Role of CD86 Polymorphisms (rs1129055, rs17281995) in Risk of Cancer: Evidence from a Meta-Analysis

**DOI:** 10.1371/journal.pone.0109131

**Published:** 2014-11-04

**Authors:** Peiliang Geng, Xiaoxin Zhao, Lisha Xiang, Yunmei Liao, Ning Wang, Juanjuan Ou, Ganfeng Xie, Chen Liu, Jianjun Li, Hongtao Li, Rui Zeng, Houjie Liang

**Affiliations:** Department of Oncology and Southwest Cancer Center, Southwest Hospital Third Military Medical University, Chongqing, China; University of North Carolina School of Medicine, United States of America

## Abstract

**Background and Purpose:**

Previous studies concerning the role of *CD86* polymorphisms (rs1129055 and rs17281995) in cancer fail to provide compelling evidence. The aim of this study was to investigate the role of common polymorphisms in the risk of cancer by meta-analysis.

**Methods:**

By using the search terms Cluster of Differentiation 86/*CD86*/B7-2/polymorphism/polymorphisms/cancer, we searched PubMed, Embase, CNKI, and Wanfang and identified four studies for rs1129055 (2137 subjects) and rs17281995 (2856 subjects) respectively. Cancer risk was estimated by odds ratio (OR) and 95% confidence interval (95% CI).

**Major Findings:**

Overall, we observed significant reduced risk of cancer in relation to rs1129055. Compared with the individuals with AA genotype, the individuals with GG genotype appeared to have 62% decreased risk to develop cancer (GG versus AA: OR, 0.62; 95% CI, 0.49–0.79; P_het.,_ 0.996). Similar effects were indicated in the G versus A allele model and the GG versus GA+AA genetic model (OR, 0.83; 95% CI, 0.74–0.93; P_het.,_ 0.987; OR, 0.63; 95% CI, 0.50–0.79; P_het.,_ 0.973). In addition, we found genotypes of rs17281995 had a major effect on overall cancer risk (CC versus GG: OR, 2.38; 95% CI, 1.43–3.95; P_het.,_ 0.433; C versus G: OR, 1.23; 95% CI, 1.06–1.43; P_het.,_ 0.521; CC versus GC+GG: OR, 2.38; 95% CI, 1.45–3.93; P_het.,_ 0.443). The association was also observed in Caucasians and colorectal cancer. No obvious publication bias was detected in this meta-analysis.

**Conclusions:**

These data reveal that rs1129055 may have protective effects on cancer risk in Asians and that rs17281995 is likely to contribute to risk of cancer, particularly colorectal cancer in Caucasians.

## Introduction

Multiple mechanisms involved in cancer have been extensively explored. But the continually increasing global burden of cancer may reflect the presently incomplete knowledge of the pathogenesis and aetiology mechanisms [1]. Inflammation, as one of these mechanisms, plays a promotive role in several biological capabilities including sustaining proliferative signaling, evading growth suppressors, resisting cell death, enabling replicative immortality, inducing angiogenesis, and activating invasion and metastasis which are required in the multistep development of human cancers [2]. Inflammatory activation has long been known as a contributing factor for cancer promotion and progression due to the potentiality of impairing the maintenance of tissue homeostasis and repair [3].

Cluster of Differentiation 86 (CD86, also known as B7-2) residing on antigen-presenting cells is a co-stimulatory molecule. It is important for autoimmunity, transplantation, and tumor immunity [4], [5]. Variation of *CD86* may make the immune cells dysfunctional and cause subsequent systemic inflammatory responses [6]. *CD86* functions as a key mediator of the activation of T cell in immune response [7]. Lack of *CD86* could lead to T cell inactivation and nonresponse to tumor cells, and thereby allows malignant progression of cancer [8].

The *CD86* gene on chromosome 3q21 is comprised of eight exons. Two most common polymorphisms, rs1129055 (+1057 G>A) and rs17281995 (+2379G>C), located in exon 8 and 3′untranslated region regulatory domain respectively, have a modulating role in the level of protein kinase C phosphorylation of *CD86* cytoplasmic tail [9]. Several population-based case-control studies have been initiated to independently investigate their roles in various cancers [10]–[13]. But a definitive role is yet to be established. Therefore, it is of great importance to comprehensively understand the polymorphisms and their functional effects to provide novel insights in the field of cancer pathophysiology. In the present study, we targeted rs1129055 and rs17281995 of the *CD86* gene and performed a meta-analysis with an aim to present compelling statistical evidence for their genetic predisposition to cancer.

## Materials and Methods

### Identification and eligibility of relevant studies

By using the search terms ‘Cluster of Differentiation 86’, ‘CD86’, ‘B7-2’, ‘polymorphism’, ‘polymorphisms’, and ‘cancer’ in combination and in isolation, we conducted a bibliography search in English databases (PubMed, Embase) as well as Chinese databases (CNKI, Wanfang), to identify all publications looking at the topic in the present study. The search was completed on November 30, 2013. We imposed no limits on language. In case of missing usable raw data, we also reviewed the references quoted in original articles.

Studies that met all of the following criteria were considered in the meta-analysis:

- Authors must recruit human cancer cases and well-matched controls;
- The publication must concentrate on the association of *CD86* polymorphisms (rs1129055 and rs17281995) and cancer risk;
- Authors had to present complete data to estimate an odds ratio (OR) with 95% confidence interval (CI);
- The study must be published on line before June 30, 2013;
- The study used an independent case population without a subsequent update; if, however, the case panel was expanded, the study with a larger number of subjects was included.

Two authors independently searched the databases and then selected the studies matching the inclusion criteria as listed above.

### Data extraction

Based on a consensus reached previously, two independent authors extracted data and recorded the characteristics of each study as follows: first author, year of publication, study country, source of controls, ethnicity, genotyping method, genetic and allele frequency between cases and controls, and type of cancer. If a single study investigated two independent case-control groups, we treated separately and classified them into Caucasian or Asian.

### Statistical analysis

Cancer risk [odds ratio (OR) and 95% confidence interval (95% CI)] in relation to *CD86* polymorphisms was assessed for each study. We first performed comparisons among all subjects, followed by stratification analyses according to gender (female and male) for rs1129055, and by cancer type (colorectal cancer and pancreatic cancer) and ethnicity (Caucasian and Asian) for rs17281995.

The χ^2^-based Q-statistic test was used to detect between-study heterogeneity that arose from methodological or clinical dissimilarity across studies [14]. Heterogeneity was considered significant when P<0.10. Values from each study were summarized using fixed-effect model with the Mantel-Haenszel method when the studies were not statistically heterogeneous; otherwise random-effect model derived from DerSimonian and Laird method was used to combine the results [15], [16]. Significance of the pooled ORs was checked by the Z-test and it reached the significant level when P<0.10. Forest plots were used to illustrate the results of included studies. Sensitivity analysis was performed by sequentially deleting each single study and recalculating the ORs. Funnel plot together with the Egger’s test were used to examine potential publication bias in the meta-analysis [17], [18]. χ^2^ test was applied to check the extent of departure from Hardy-Weinberg equilibrium (HWE) for genotype distribution in controls. All analyses were done using Stata 12.0 (Stata Corporation, College Station, TX). The level of significance was set at P<0.10.

## Results

### Characteristics of studies

**Figure 1:**
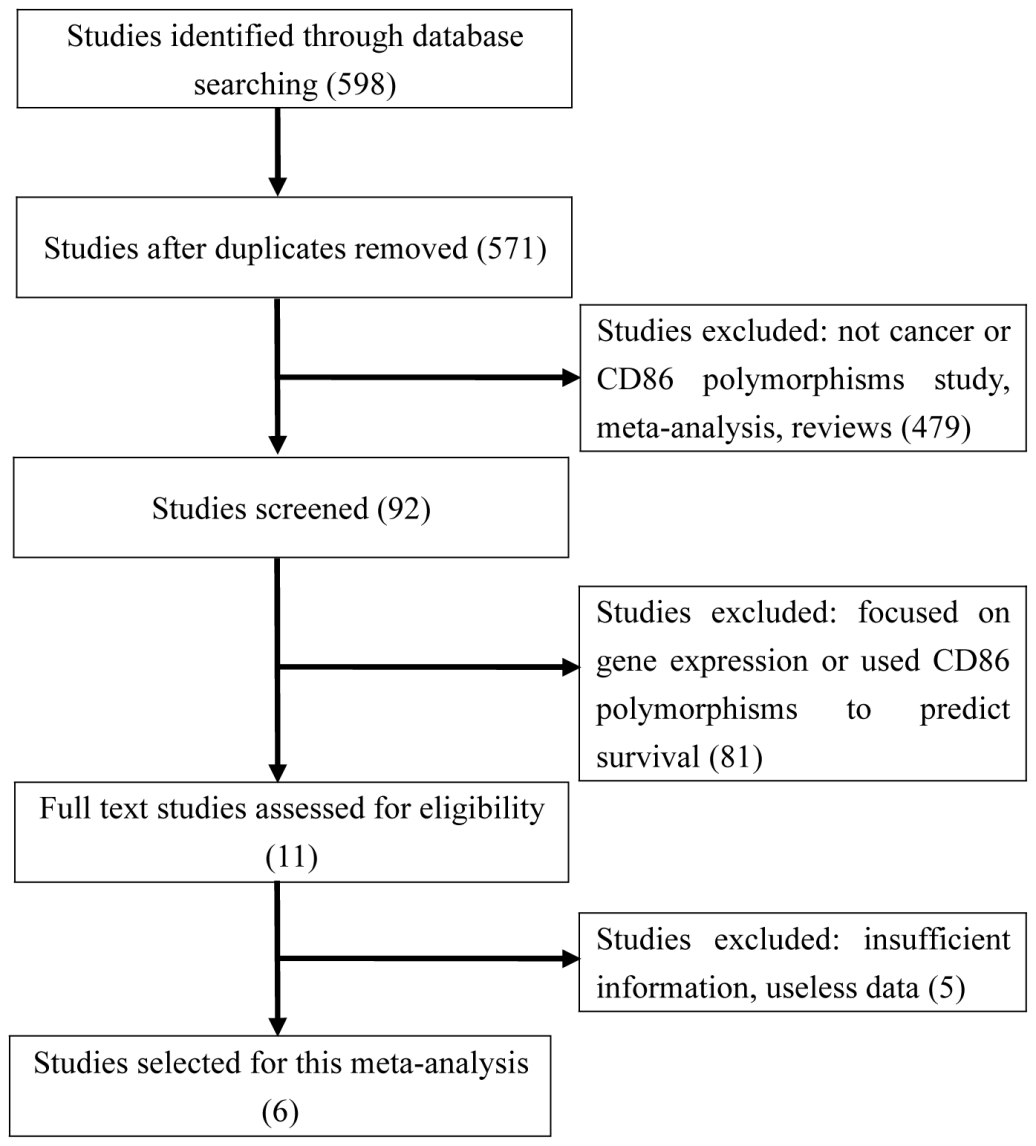
Flow diagram of studies identification.

We finally identified six publications [8], [10]–[13], [19] of *CD86* polymorphisms and cancer risk. The detailed process of study identification is presented in Figure 1. We summarized the main characteristics of each study in Table 1.

**Table 1:** Main characteristics of all studies included in the meta-analysis.

First author	Year	Area	Ethnicity	Single nucleotide polymorphism studied	Cancer type	No. of cases	No. of controls	Genotyping method	Control source
Pan	2010	China	Asian	rs1129055	CRC	273	292	PCR-RFLP	PCC
Wang	2011	China	Asian	rs1129055	OS	205	216	PCR-RFLP	PCC
Wang	2012	China	Asian	rs1129055	ES	158	212	PCR-RFLP	PCC
Xiang	2012	China	Asian	rs1129055	PC	369	412	PCR-RFLP	PCC
Landi	2011	Czech	Caucasian	rs17281995	CRC	660	556	TaqMan	HCC
Landi	2011	Spain	Caucasian	rs17281995	CRC	304	255	NA	NA
Xiang	2012	China	Asian	rs17281995	PC	369	412	PCR-RFLP	PCC
Azimzadeh	2013	Iran	Caucasian	rs17281995	CRC	150	150	TaqMan	PCC

CRC, colorectal cancer; OS, osteosarcoma; ES, Ewing’s Sarcoma; PC, pancreatic cancer; PCR-RFLP, polymerase chain reaction-restriction fragment length polymorphism; NA, not available; PCC, population-based case-control study; HCC, hospital-based case-control study.

For rs1129055, four publications were included and different cancers (colorectal cancer, osteosarcoma, Ewing’s Sarcoma, pancreatic cancer) were investigated. All of the case-control studies were conduced in China. In addition, they selected Chinese as study subjects, used population-based controls and applied PCR-RFLP (polymerase chain reaction-restriction fragment length polymorphism) in genotype determination.

However, for rs17281995, several study countries were involved, varying from Czech to Iran. Among the included studies, two types of cancer (three on colorectal cancer and one on pancreatic cancer) were investigated, and both Caucasian and Asian subjects were used.

All genotype distribution in controls was in accordance with HWE with the exception of one study for rs17281995 [13].

### Sensitivity analyses

In order to detect the impact of each dataset on the summary results, we conducted sensitivity analysis by sequentially deleting the single studies involved in the meta-analysis. The ORs were not materially modified by the sequential removals, implicating our results were stable and credible (data not shown).

### Publication bias

**Figure 2:**
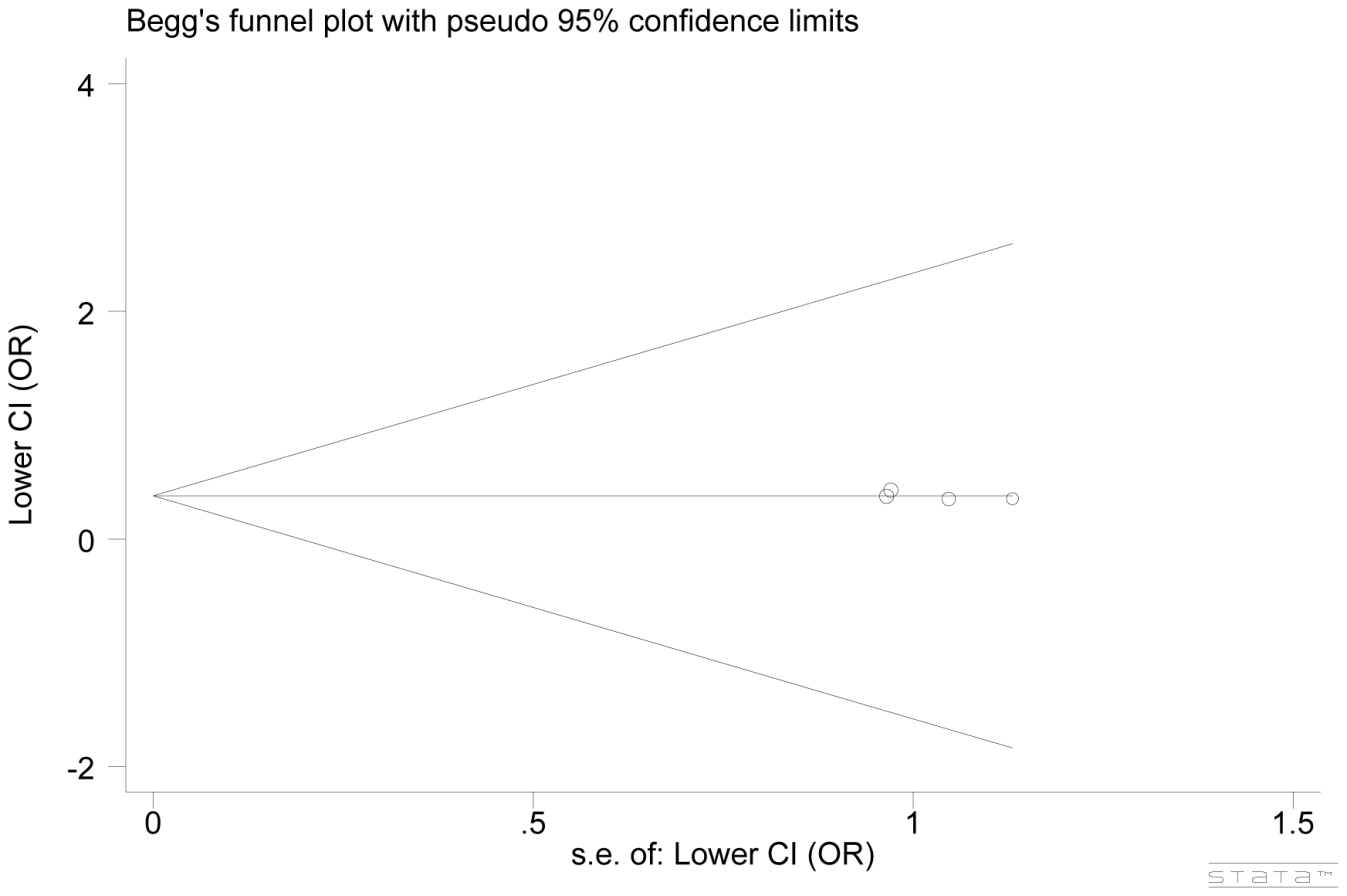
Funnel plot of publication bias analysis for the associations between CD86 polymorphism and cancer risk.

Publication bias was checked by Begg’s funnel plot and Egger’s test. For all genetic models of *CD86* polymorphisms, the shape of funnel plots revealed little evidence of obvious asymmetry. Statistically supportive evidence that there was no significant publication bias was further presented in the Egger’s test (Figure 2: funnel plot for rs1129055, P_Begg_ = 0.731, P_Egger_ = 0.331 under GG versus AA;).

### Cancer risk associated with rs1129055

**Figure 3:**
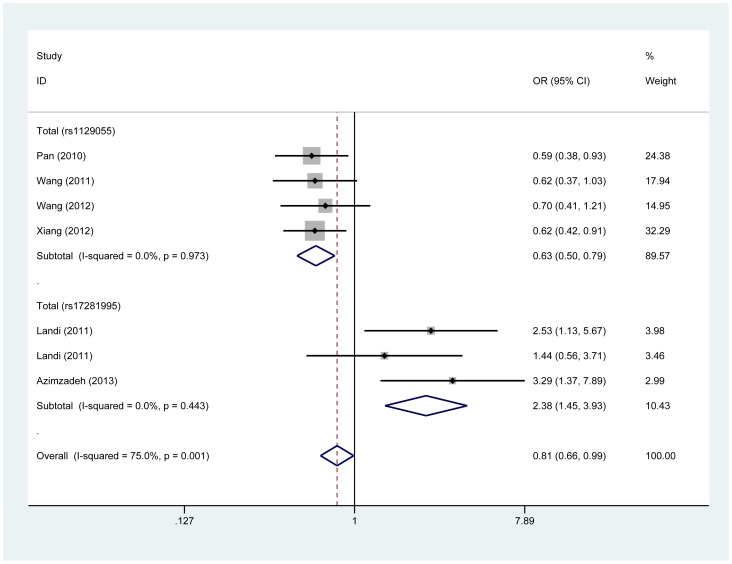
Forest plot of estimates of the odds ratios (ORs) for CD86 polymorphisms in cancer under GG versus GA+AA for rs1129055 and CC versus GC+GG for rs17281995. The squares and horizontal lines correspond to ORs and 95% CIs of specific study, and the area of squares reflects study weight (inverse of the variance). The diamond represents the pooled ORs and its 95% CIs.

By combining four studies of rs1129055, we yielded 2137 subjects for this meta-analysis. Overall, we observed significant reduced risk of cancer in relation to rs1129055. Compared with the individuals with AA genotype, the individuals with GG genotype appeared to have 62% decreased risk to develop cancer (GG versus AA: OR, 0.62; 95% CI, 0.49–0.79; P_het.,_ 0.996). Similar effects were also indicated in the G versus A allele model and the GG versus GA+AA genetic model (OR, 0.83; 95% CI, 0.74–0.93; P_het.,_ 0.987; OR, 0.63; 95% CI, 0.50–0.79; P_het.,_ 0.973) (Figure 3). On the contrary, when we carried out comparisons between female and male, none of the genetic models showed effect modification of cancer risk. The studies were statistically homogeneous under all genetic models (Figure 2, Table 2).

**Table 2:** Results of meta-analysis for CD86 polymorphisms and cancer.

rs1129055 Study group	GG versus AA		G versus A		GA versus AA		GG+GA versus AA		GG versus GA+AA	
	OR (95% CI)	P	OR (95% CI)	P	OR (95% CI)	P	OR (95% CI)	P	OR (95% CI)	P
Total	0.62 (0.49, 0.79)	0.996	0.83 (0.74, 0.93)	0.987	0.90 (0.78, 1.05)	0.963	0.89 (0.78, 1.01)	0.980	0.63 (0.50, 0.79)	0.973
Gender^a^	0.76 (0.46, 1.25)	0.472	0.88 (0.71, 1.10)	0.469	0.89 (0.67, 1.18)	0.699	0.90 (0.69, 1.16)	0.671	0.82 (0.51, 1.32)	0.645
rs17281995 Study group	CC versus GG		C versus G		GC versus GG		CC+GC versus GG		CC versus GC+GG	
	OR (95% CI)	P	OR (95% CI)	P	OR (95% CI)	P	OR (95% CI)	P	OR (95% CI)	P
Total	2.38 (1.43, 3.95)	0.433	1.23 (1.06, 1.43)	0.521	1.12 (0.95, 1.33)	0.769	1.69 (0.99, 1.37)	0.711	2.38 (1.45, 3.93)	0.443
Cancer type										
CRC	2.38 (1.43, 3.95)	0.433	1.25 (1.07, 1.47)	0.358	1.12 (0.93, 1.35)	0.569	1.17 (0.98, 1.40)	0.506	2.38 (1.45, 3.93)	0.443
PC	–	–	1.14 (0.78, 1.67)	–	1.14 (0.77, 1.69)	–	1.14 (0.77, 1.69)	–	–	–

a results for female versus male;

P, p value for heterogeneity test.

### Cancer risk associated with rs17281995

Next, we assessed the effects of rs17281995 on cancer risk based on three publications, including four independent case-control populations with a total of 2856 participants. We found genotypes of rs17281995 had a major effect on overall cancer risk (CC versus GG: OR, 2.38; 95% CI, 1.43–3.95; P_het.,_ 0.433; C versus G: OR, 1.23; 95% CI, 1.06–1.43; P_het.,_ 0.521; CC versus GC+GG: OR, 2.38; 95% CI, 1.45–3.93; P_het.,_ 0.443).

In the stratified analysis by cancer type, the results showed significant association with colorectal cancer under the CC versus GG, C versus G, and CC versus GC+GG models (OR, 2.38; 95% CI, 1.43–3.95; P_het.,_ 0.433; OR, 1.25; 95% CI, 1.07–1.47; P_het.,_ 0.358; OR, 2.38; 95% CI, 1.45–3.93; P_het.,_ 0.433). Consistent with the results in the analysis of cancer type, obviously increased risk was also found in Caucasian population (Table 2).

## Discussion

The *CD86* is a single-copy gene in humans. It has a critical role in the regulation of T cell responses, including T cell activation and tolerance, through the CD28/CTLA-4 pathway [5], [20]. *CD86* has been detected in immune system cells and is involved in the pathogenesis of a broad range of inflammation-associated diseases, such as asthma and related allergic disorders (21) [21], pancytopenia [22], leprosy [23], sepsis [24] and liver transplantation [25]. However, in addition to the aforementioned diseases, the exact role of *CD86* in cancer susceptibility also requires to be followed up by replication and functional studies to determine a definitive causative role.

Currently, considerable effort is underway to connect human phenotypes with variation at the DNA level. It is believed that human genetic variations are able to cause phenotypic differences between individuals and most of these abnormalities should be dominantly assigned to single nucleotide polymorphisms [26] Hence, to make clear the biological function of these polymorphisms in malignant diseases, especially in various cancers, further independent large investigations are essential to provide useful insights and expand the current knowledge.

rs1129055 (+1057 G>A) and rs17281995 are two widely investigated polymorphisms in the *CD86* gene. Since the first report on the association of rs17281995 and risk of sporadic colorectal cancer in Caucasians was published in 2008 [27], a number of case-control association studies have been followed up [10]–[13]. These studies nevertheless used a relatively insufficient sample size and focused on different types of cancer, which may lead to a false-positive or negative-positive conclusion. Moreover, lack of meta-analysis on *CD86* polymorphisms and cancer risk led us to perform the present study to validate the results.

In our study, we investigated two polymorphisms, namely rs1129055 and rs17281995. From the meta-analysis results, we found the genotypes of rs1129055, especially the GG genotype, were associated with obviously deceased risk of cancer. Further analysis between female and male revealed that females were not more likely to develop cancer as compared with males. On the contrary, for rs17281995, we observed significantly increased risk of overall cancer. The association was also observed in subgroups by ethnicity and cancer type: for Caucasians and for colorectal cancer under CC versus GG, C versus G, and CC versus GC+GG models.

For rs1129055, there are several similarities among the four studies: all subjects were Asians, source of controls was uniformly population-based, and the studies used the same genotyping method. This may be the major reason for the high homogeneity across studies. For rs17281995, significant association was only observed in Caucasian population, but not in Asian population. A reasonable interpretation is the marked difference in the study size (2075 versus 781), because a lager sample is more likely to result in a conclusion that is close to the real association.

Several factors should be considered when interpreting our results. Firstly, even though we have summarized all data on *CD86* polymorphisms and cancer risk, the total sample still needs further expansion. Secondly, only Asian population is involved in the analysis of rs1129055, and most studies of rs17281995 are for Caucasian population. Therefore, it is nice to include more studies with various ethnic groups considered to identify their definitive roles in varying populations. Thirdly, as cancer is a multifactorial disease, thus more confounding factors such as age, tobacco use, alcohol consumption remain to be investigated. Meanwhile, multiple strong points in our meta-analysis should be addressed. The first strong point is that this is the first study examining the associations between *CD86* polymorphisms and cancer risk to date, and the associations were assessed using a rigorous method. The second strong point refers to the stability and reliability of our results. Because the included studies are not statistically heterogeneous and there is no evidence for significant publication bias in the literature.

In summary, the present meta-analysis supported a significant association between rs1129055 genotypes and decreased risk of cancer in Asians. However, obviously increased cancer risk was observed in the overall analysis as well as subgroup analyses by ethnicity and cancer type for rs17281995. Whether *CD86* polymorphisms could serve as a biomarker for genetic susceptibility to cancer requires to be further validated in future.

## Supporting Information

Checklist S1 (DOC)
